# Incorporation of membrane-anchored flagellin or *Escherichia coli* heat-labile enterotoxin B subunit enhances the immunogenicity of rabies virus-like particles in mice and dogs

**DOI:** 10.3389/fmicb.2015.00169

**Published:** 2015-03-03

**Authors:** Yinglin Qi, Hongtao Kang, Xuexing Zheng, Hualei Wang, Yuwei Gao, Songtao Yang, Xianzhu Xia

**Affiliations:** ^1^College of Veterinary Medicine, Jilin UniversityChangchun, China; ^2^Institute of Military Veterinary Medicine, Academy of Military Medical ScienceChangchun, China; ^3^College of Veterinary Medicine, South China Agricultural UniversityGuangzhou, China

**Keywords:** rabies virus, virus-like particle, flagellin, LTB, rabies vaccine

## Abstract

Rabies remains an important worldwide public health threat, so safe, effective, and affordable vaccines are still being sought. Virus-like particle-based vaccines targeting various viral pathogens have been successfully produced, licensed, and commercialized. Here, we designed and constructed two chimeric rabies virus-like particles (cRVLPs) containing rabies virus (RABV) glycoprotein (G), matrix (M) protein, and membrane-anchored flagellin (EVLP-F) or *Escherichia coli* heat-labile enterotoxin B subunit (EVLP-L) as molecular adjuvants to enhance the immune response against rabies. The immunogenicity and potential of cRVLPs as novel rabies vaccine were evaluated by intramuscular vaccination in mouse and dog models. Mouse studies demonstrated that both EVLP-F and EVLP-L induced faster and larger virus-neutralizing antibodies (VNAs) responses and elicited greater numbers of CD4^+^ and CD8^+^ T cells secreting IFN-γ or IL-4 compared with a standard rabies VLP (sRVLP) containing only G and M. Moreover, cRVLPs recruited and/or activated more B cells and dendritic cells in inguinal lymph nodes. EVLP-F induced a strong, specific IgG2a response but not an IgG1 response, suggesting the activation of Th1 class immunity; in contrast, Th2 class immunity was observed with EVLP-L. The significantly enhanced humoral and cellular immune responses induced by cRVLPs provided complete protection against lethal challenge with RABV. Most importantly, dogs vaccinated with EVLP-F or EVLP-L exhibited increased VNA titers in sera and enhanced IFN-γ and IL-4 secretion from peripheral blood mononuclear cells. Taken together, these results illustrate that when incorporated into sRVLP, membrane-anchored flagellin, and heat-labile enterotoxin B subunit possess strong adjuvant activity. EVLP-F and EVLP-L induce significantly enhanced RABV-specific humoral and cellular immune responses in both mouse and dog. Therefore, these cRVLPs may be developed as safe and more efficacious rabies vaccine candidate for animals.

## INTRODUCTION

Rabies is an ancient zoonotic infectious disease that is distributed worldwide. The causative agent is rabies virus (RABV), which mainly infects neurons and proceeds to the central nervous system (CNS), causing severe and fatal viral encephalitis in animals and humans (Chopy et al., 2011). Despite great progress in the development of rabies vaccines, more than 55,000 humans die from rabies around the world each year, with most of these deaths occurring in developing countries (Ge et al., 2011).

Virus-like particles (VLPs) present to the immune system like natural viruses and initiate innate immunity, which eventually results in adaptive and inflammatory responses to control infection from various viruses (Jackson, 2002). VLPs have greater potential as vaccine candidates than soluble protein because of their ability to activate antigen-presenting cells (APCs) when administered via multiple routes (Bright et al., 2007; Sailaja et al., 2007; Raghunandan, 2011). Previously, we constructed a standard rabies VLP (sRVLP) that consists of the RABV glycoprotein (G) and matrix protein (M) and evaluated its immunogenicity in mice and dogs (Hong-Tao Kang et al., submitted for publication). To improve the immunogenicity of sRVLP and to develop a more effective rabies vaccine candidate, we further modified the sRVLP to enhance the immune responses against rabies.

The innate immune response is the host’s first line of defense against infection and is a crucial step for eliciting the adaptive immune response. Flagellin is a ligand for TLR5, and triggering of the TLR5 signaling pathway ultimately results in the activation and recruitment of B and T cells, secretion of inflammatory cytokines, and expression of major histocompatibility complex (MHC) and costimulatory molecules on APCs (Hayashi et al., 2001; Means et al., 2003; Tallant et al., 2004; Feuillet et al., 2006). Flagellin acts as an adjuvant even at a very low dose and does not induce IgE responses, and the pre-existing immune responses targeting flagellin do not affect its potency (Ben-Yedidia and Arnon, 1998; Honko et al., 2006; Weimer et al., 2009). The mixtures of soluble flagellin plus antigen do not induce specific immune responses, and the fusion protein of antigen with flagellin may be necessary for the production of antigen-specific immune responses (Wang et al., 2008). The flagellin-antigen fusion has been demonstrated to provide better adjuvant efficacy irrespective of the site of introduction at the N or C termini or within the hypervariable regions of the protein (Wang et al., 2010; Bates et al., 2011). *Escherichia coli* heat-labile enterotoxin B subunit (LTB) is non-toxic and mediates high-affinity binding to the galactosyl-N-acetylgalactosamylsialyl-galactosylglucosylceramide (GM1) ganglioside receptor on the surfaces of all mammalian cells (Spangler, 1992). LTB has immunomodulatory properties that stimulate both systemic and mucosal immune responses and promote the induction of Th1 and Th2 responses (Hagiwar et al., 2001). Therefore, LTB is a potent vaccine adjuvant and immune modulator in a range of disease models, functioning by inducing B cell activation and CD8^+^ T cell apoptosis and modulating monocyte function (Zhang et al., 2010; Donaldson et al., 2011, 2013; Sun et al., 2013). More importantly, flagellin and LTB are well known as powerful mucosal adjuvants when co-administered with antigen or administered in the form of an antigen fusion. Flagellin stimulates the secretion of the CCL20 chemokine from epithelial cells, triggering DC chemotaxis (Sierro et al., 2001). LTB vaccination improves antigen uptake by binding to the GM1 ganglioside on intestinal enterocytes (Liu et al., 2011; Wang et al., 2012).

In the present study, we designed and constructed two chimeric rabies VLPs (cRVLPs) containing G and M of the RABV Evelyn-Rokitnicki-Abelseth (ERA) strain, and membrane-anchored forms of flagellin or LTB in insect cells. We confirmed the expression of flagellin and LTB and the assembly integrity of the two cRVLPs and then, evaluated the immune responses induced by these cRVLPs using mouse and dog models. Finally, we investigated the ability of the cRVLPs to protect the animals against a lethal challenge with RABV.

## MATERIALS AND METHODS

### CELL LINE AND VIRUS STRAINS

Sf9 cells were cultured with serum-free SF900II medium (Life technologies, San Diego, CA, USA) in suspension in flasks at 27°C at a speed of 120 rpm. HuNPB_3_ is a RABV street strain that was isolated from a pig that died of rabies in the Hunan Province of China in 2006 and stored in our laboratory. The RABV ERA (Accession NO. EF206707) strain was obtained from the China Veterinary Culture Collection.

### CONSTRUCTION OF RECOMBINANT PLASMIDS

Recombinant plasmids were generated by fusing the genes encoding the signal peptide (SP) from honeybee mellitin and the transmembrane (TM) and cytoplasmic tail (CT) regions from the G of the RABV ERA in frame to the 5′ and 3′ ends of the flagellin or LTB genes, respectively (Wang et al., 2008). The SP of honeybee mellitin is known to improve glycoprotein cell surface expression in insect cells (Li et al., 1994; Wang et al., 2007). The TM-CT was used as a membrane anchor sequence for the incorporation of modified flagellin or LTB. All primers used in the present study are listed in **Table 1**. The MSP-FL1 fragment (containing part of the mellitin SP and flagellin) was amplified from pMD-FL (containing the full-length flagellin gene, GenBank accession no. NP_460912; a kind gift from Dr. Hualei Wang) using primers MSP-FLF1 and MSP-FLR. The MSP-FL2 fragment (containing the integral mellitin SP and flagellin) was amplified from MSP-FL1 using primers MSP-FLF2 and MSP-FLR and was cloned into the BamHI/EcoRI sites of the pFastBac Dual vector under the control of the polyhedrin (PH) promoter, resulting in plasmid pFBD-MF. The EG-TMCT fragment (containing the TM and CT regions of the G gene) was amplified from cDNA of ERA using primers EG-TMCTF and EG-TMCTR and inserted into the pFBD-MF construct using the EcoRI/HindIII sites, resulting in pFBD-MFG. Then, using pFBD-MFG as a template and MFGF and MFGR as primers, MFG-XN was cloned into the XhoI/NheI sites of pFBD-MFG under the control of the p10 promoter, creating pFBD-2MFG. pFBD-2MLG contains two identical LTB genes that consist of the mellitin SP, LTB, and TM-CT regions and was constructed in the same way as pFBD-2MFG but using pMD-ML (containing the mellitin SP and LTB genes, GenBank accession no. AB011677.1; a kind gift from Dr. Zhiguang Ren) as the template with the primer sets MLF and MLR, EG-TMCTF and EG-TMCTR, and MLGF and MLGR.

**Table 1:** Sequences of primers used in present study.

Primer	Sequence (5′-3′)	Restriction enzyme site
MSP-FLF1	**TTTTATGGTCGTGTACATTTCTTACATCTATGCGGCCGCT**ATGGCACAAGTCATTAATACA	
MSP-FLF2	CCCCGGATCC**ATGAAATTCTTAGTCAACGTTGCCCTTGTTTTTATGGTCGTGTACATTTC**	BamH I
MSP-FLR	CCCGAATTCACGCAGTAAAGAGAGGACGTTTT	EcoR I
EG-TMCTF	CCCCGAATTCTATGTATTACTGAGTGCAGG	EcoR I
EG-TMCTR	TTTTAAGCTTTCACAGTCTGGTCTCACCCC	Hind III
MFGF	CCCCTCGAG**ATGAAATTCTTAGTCAACGTTGCCCTTGTTTTTATGGTCGTGTACATTTC**	Xho I
MFGR	TTTTGCTAGCTCACAGTCTGGTCTCACCCC	Nhe I
MLF	CCCGGATCCATGAAATTCCTGGTGAACGTC	BamH I
MLR	CCCGAATTCGTTCTCCATGGAAATAGCAGC	EcoR I
MLGF	GGGCTCGAGATGAAATTCCTGGTGAACGTCGCTCT	Xho I
MLGR	AAAGCTAGCTTACAGGCGGGTCTCGCCACCGGACT	Nhe I

*The sequences of restriction enzyme sites are underlined*.

*The sequences of SP from honeybee mellitin are bolded*.

### GENERATION OF rBVs And Preparation OF cRVLPs

The rBVs rpFBD-2MFG and rpFBD-2MLG expressing flagellin and LTB, respectively, were generated using the Cellfection®;II Reagent (Life technologies, San Diego, CA, USA) according to the manufacturer’s instructions. The rBVs rpFBD-2COG and rpFBD-2COM expressing the G and M genes of the RABV ERA strain, respectively, were generated in a previous study (Hong-Tao Kang et al., submitted for publication). EVLP-F (the cRVLP containing flagellin) was produced by co-infecting Sf9 cells with rpFBD-2COG, rpFBD-2COM, and rpFBD-2MFG. EVLP-L (the cRVLP containing LTB) was produced by co-infecting Sf9 cells with rpFBD-2COG, rpFBD-2COM, and rpFBD-2MLG. EVLP is the sRVLP containing only G and M of RABV ERA and was produced as described previously (Hong-Tao Kang et al., submitted for publication). All VLPs were harvested at day 5 post-infection and were centrifuged to remove cell debris, pelleted, and purified through a 20–40–60% discontinuous sucrose gradient. The VLP bands between 40 and 60% sucrose were collected.

### WESTERN BLOT

The cRVLPs were analyzed by SDS-PAGE under denaturing conditions before transferring the proteins onto a nitrocellulose membrane (Whatman, Kent, UK) for Western blotting with anti-rabies G (Millipore, Boston, MA, USA), anti-flagellin (BioLegend, San Diego, CA, USA), or anti-LTB (Abcam, Cambridge, MA, USA) mouse monoclonal antibodies or with rabbit serum against M.

### ELECTRON AND IMMUNOELECTRON MICROSCOPY

The cRVLPs were applied to grids, stained with 1% sodium phosphotungstate, and then observed using transmission electron microscopy. The cRVLPs were also bound to formvar-coated grids, incubated with mouse anti-flagellin or anti-LTB antibodies, and then incubated with gold-labeled goat anti-mouse IgG antibody (Sigma-Aldrich, Saint Louis, MO, USA). Finally, the grids were negatively stained and observed.

### IMMUNIZATION STUDIES

Eight-week-old female BALB/c mice and 3-month-old beagles were purchased from the Changchun Institute of Biological Products Co., Ltd, China. Mice and dogs were randomized into four groups by drawing numbers out of a hat. Mice were oxvaccinated intramuscularly (i.m.) in the gastrocnemius muscle with 10 μg/mouse of EVLP, EVLP-F, or EVLP-L. Dogs were inoculated i.m. in the gastrocnemius muscle with 0.15 mg/dog of the same VLPs. All mice and dogs received a second identical vaccination 2 weeks after the initial immunization. One group was treated with PBS as a control.

### ANTIBODY RESPONSES

Blood samples from mice were collected at weeks 1, 2, 4, and 6 by retro-orbital plexus puncture. All dogs were bled from the vein in the front leg at weeks 4 and 6. Virus-neutralizing antibody (VNA) was measured using fluorescent antibody virus neutralization (FAVN; Cliquet et al., 1998). RABV-specific antibody responses for IgG, IgG1, IgG2a, IgG2b, IgG3, and IgM in mouse sera were determined using an enzyme-linked immunosorbent assay (ELISA; Wang et al., 2008; Quan et al., 2011).

### IFN-γ AND IL-4 ENZYME-LINKED IMMUNOSPOT (ELISpot) ASSAYS

Splenocytes from mice and peripheral blood mononuclear cells (PBMCs) from dogs were isolated at 2 weeks after the second immunization and stimulated with inactivated ERA at a final concentration of 10 μg/ml. Cells producing IFN-γ or IL-4 were identified using enzyme-linked immunospot (ELISpot) kits (Mabtech AB, Stockholm, Sweden; R&D Systems, Minneapolis, MN, USA) according to the manufacturer’s instructions. Spot-forming cells (SFCs) were counted using an automated ELISpot reader (AID ELISPOT reader-iSpot, AID GmbH, GER).

### FLOW CYTOMETRY ASSAYS FOR INTRACELLULAR CYTOKINE STAINING (ICS) AND B CELLS AND DCs IN LYMPH NODES FROM MICE

Two weeks after the second vaccination, splenocytes (1 × 10^7^ cells/ml) were isolated, cultured, and stimulated with inactivated ERA (10 μg/ml) in the presence of monensin. At 6 h post-stimulation, surface staining was performed with anti-CD4 and anti-CD8 monoclonal antibodies. The cells were permeabilized with Cytofix/Cytoperm and then intracellularly stained with anti-IFN-γ and anti-IL-4 monoclonal antibodies. Inguinal lymph node samples were collected at days 3, 6, and 9 after the first immunization. The cell suspensions were stained with anti-CD19, anti-CD40, anti-CD11c, anti-CD80, anti-CD86, anti-MHC I, and anti-MHC II monoclonal antibodies. All labeled cells were analyzed with a flow cytometer. Antibodies and reagents used in flow cytometry assays were purchased from BD Pharmingen (BD Biosciences, Franklin, VA, USA).

### CHALLENGE EXPERIMENT IN MICE

The mice were challenged with 100 times the 50% intramuscular mouse lethal dose (100 × IMLD_50_) of the RABV street strain HuNPB_3_ by i.m. injection 4 weeks after the final vaccination and were observed daily for 21 days. During the observation period, any mouse that developed clinical signs of rabies was humanely euthanized by cervical dislocation under isoflurane anesthesia.

### LABORATORY FACILITY AND ETHICS STATEMENT

All animal studies were conducted with prior approval from the Animal Welfare and Ethics Committee of the Veterinary Institute at the Academy of Military Medical Sciences under permit number SCXK-2012-017. The environment and housing facilities satisfied the National Standards of Laboratory Animal Requirements (GB 14925-2001) of China. Experiments related to the RABV street strain were conducted in a biosecurity level 3 laboratory and were approved by the Military Veterinary Research Institute of the Academy of Military Medical Sciences.

## RESULTS

### CONSTRUCTION, PURIFICATION, AND IDENTIFICATION OF cRVLPs

**FIGURE 1:**
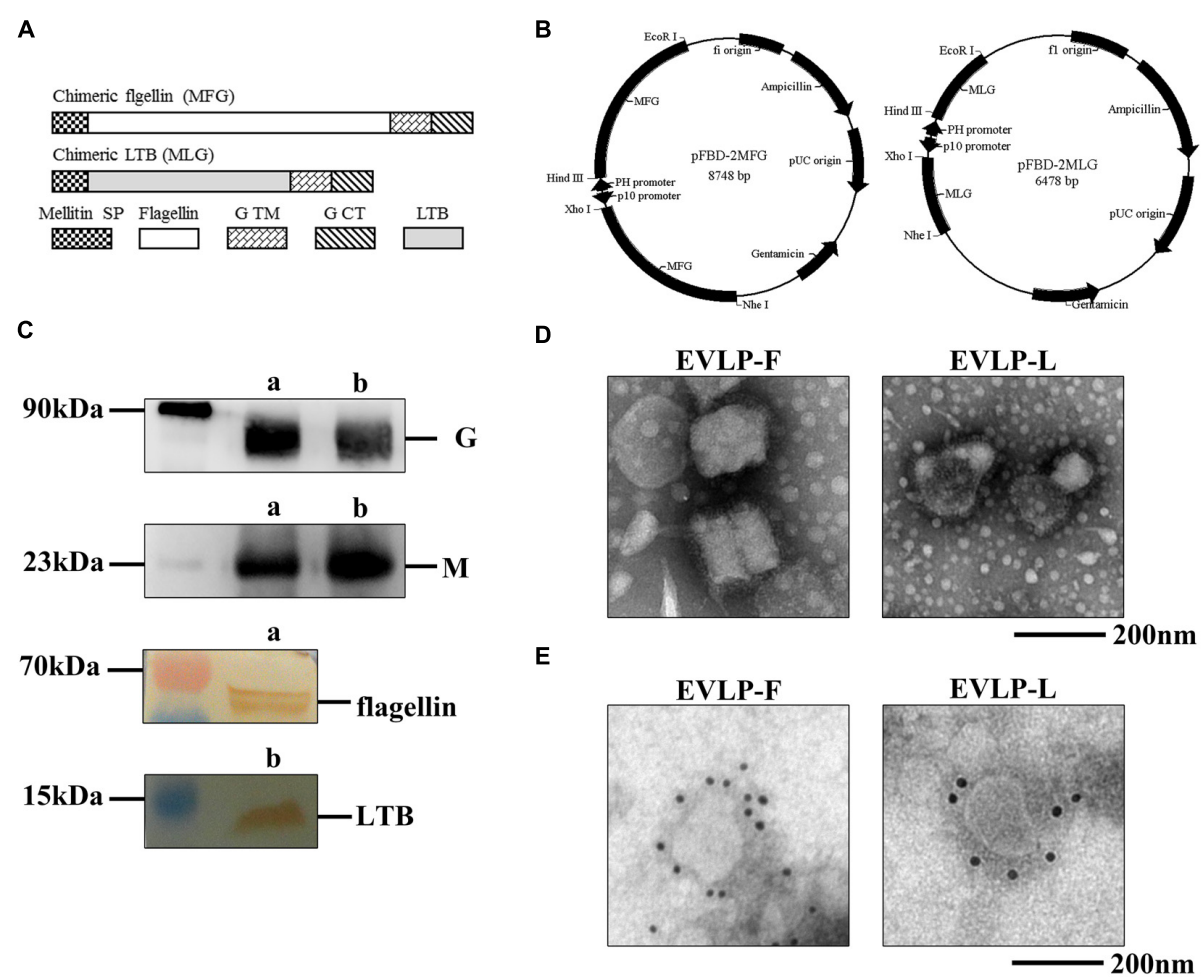
**Construction of membrane-anchored flagellin and LTB and identification of cRVLPs. (A)** Schematic diagrams for the construction of membrane-anchored flagellin and LTB. **(B)** Schematic diagrams of the recombinant plasmids pFBD-2MFG and pFBD-2MLG. **(C)** Western blot analyses of cRVLPs. EVLP-F (a) and EVLP-L (b) were incubated with mouse anti-rabies G monoclonal antibody, rabbit serum against M, mouse anti-flagellin, or mouse anti-LTB monoclonal antibodies. **(D)** Electron microscopy of cRVLPs. EVLP-F and EVLP-L were stained with 1% sodium phosphotungstate and observed by transmission electron microscopy. **(E)** Immunoelectron microscopy of cRVLPs. EVLP-F and EVLP-L were stained with anti-flagellin, or anti-LTB monoclonal antibodies followed by a gold-labeled goat anti-mouse IgG antibody.

We designed membrane-anchored forms of flagellin or LTB to enable their incorporation into sRVLP. The design strategy is shown in **Figure 1A**. Membrane-anchored flagellin and LTB were constructed by fusing them to the SP sequence from honeybee mellitin at their N termini and to the TM-CT sequence from the RABV G at their C termini. We simultaneously cloned two identical gene copies (flagellin or LTB) into one vector under the control of the PH or p10 promoter, respectively (**Figure 1B**). The two rBVs (rpFBD-2MFG and rpFBD-2MLG) were successfully recovered from transfected Sf9 insect cells.

The cRVLPs containing flagellin or LTB were produced, isolated, and purified as described in the Materials and Methods. The composition of each cRVLP was confirmed by Western blot. As shown in **Figure 1C**, we observed bands corresponding to G, M, and flagellin in EVLP-F and bands corresponding to G, M, and LTB in EVLP-L. The morphology was investigated using electron microscopy. **Figure 1D** showed that the diameters of EVLP-F and EVLP-L were approximately 180–200 nm; additionally, obvious surface spikes were observed. To determine whether flagellin or LTB were directly incorporated into sRVLPs, we performed immunoelectron microscopy. **Figure 1E** shows that multiple gold particles bound to EVLP-F when an antibody against flagellin was used, whereas anti-LTB only bound to EVLP-L. These results indicate that flagellin or LTB was incorporated onto the surface of the cRVLPs and that their incorporation did not change the morphology of the rabies VLP.

### cRVLPs CONTAINING FLAGELLIN or LTB INDUCE ENHANCED HUMORAL IMMUNE RESPONSES IN MICE

**FIGURE 2:**
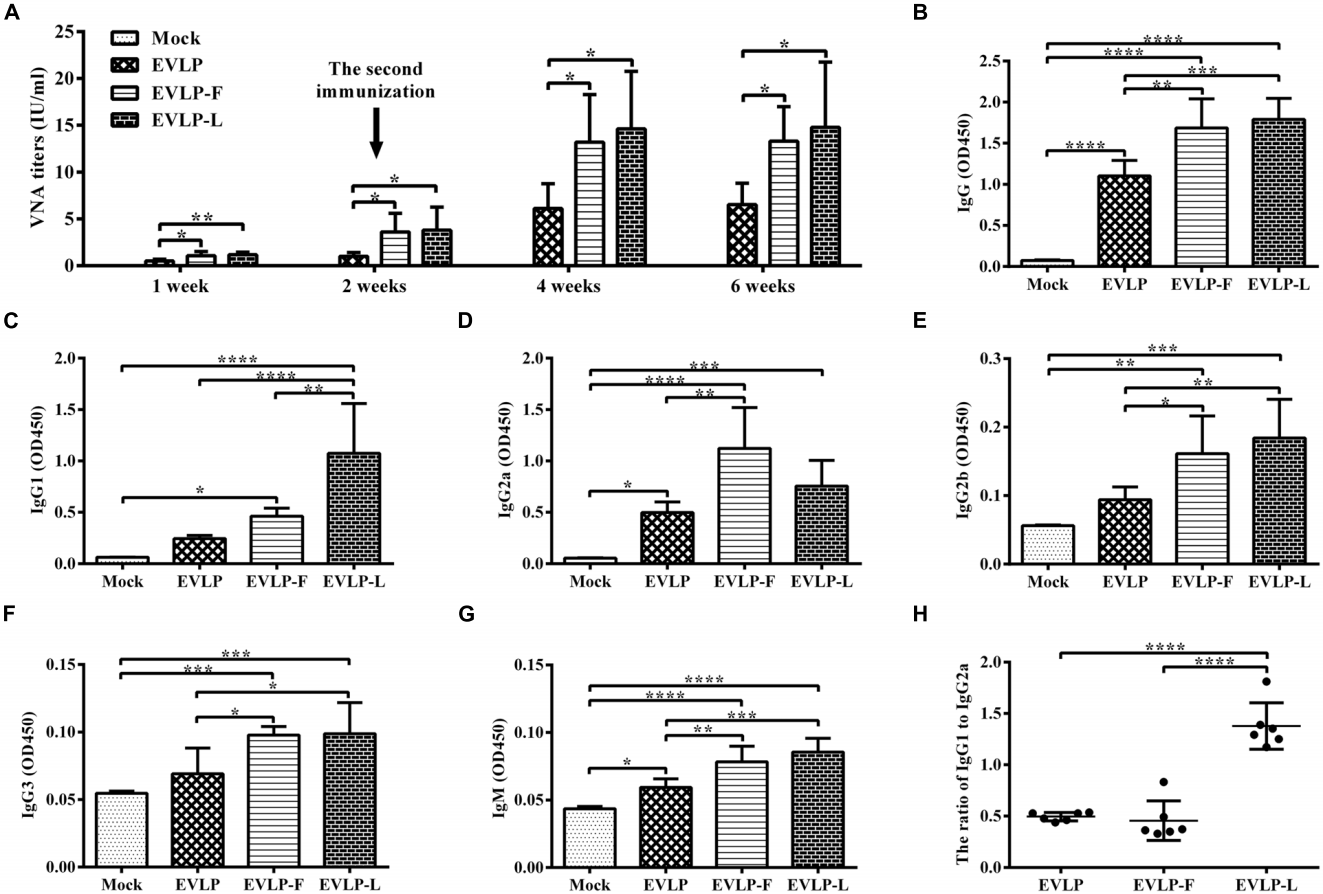
**Specific anti-RABV antibody responses in mice.** Mice were immunized twice via the i.m. route at 2-week intervals with EVLP, EVLP-F, EVLP-L, or PBS. Serum samples were then collected 1, 2, 4, and 6 weeks after the primary vaccination. **(A)** VNA titers were measured by FAVN. **(B–H)** The specific anti-RABV serum IgG and isotype responses were detected by ELISA. The serum dilution factor was 5,000. The serum IgG **(B)**, IgG1 **(C)**, IgG2a **(D)**, IgG2b **(E)**, and IgG3 **(F)** responses were determined 2 weeks after the second immunization, whereas serum IgM **(G)** responses were determined 1 week after the first immunization. The IgG1/IgG2a ratio **(H)** was measured. The data represent the mean and SD from eight mice in each group and were analyzed by one-way ANOVA (*P < 0.05, **P < 0.01, ***P < 0.001, ****P < 0.0001).

To evaluate the adjuvant effect of membrane-anchored flagellin or LTB in cRVLPs, humoral immune responses to EVLP, EVLP-F, or EVLP-L were investigated in mice. As shown in **Figure 2A**, VNA was detected in all vaccinated mice 1 week after the first dose, although the titers were relatively low. Two weeks after the first dose, the mean titers of VNA in the groups vaccinated with EVLP, EVLP-F, and EVLP-L were 1.01, 3.61, and 3.81 IU/ml, respectively. After receiving the second immunization at 2 weeks, all groups treated with VLPs showed substantially enhanced immune responses to RABV. At 4 weeks, VNA titers in the three groups listed above rose sharply to 6.12, 13.19, and 14.61 IU/ml, respectively. VNA titers remained relatively stable until 6 weeks after vaccination. Significantly higher VNA titers were measured in mice immunized with EVLP-F or EVLP-L compared with EVLP at all time points after vaccination. There were no significant differences in VNA titers between the EVLP-F and EVLP-L groups. Overall, these results indicate that mouse VNA responses to cRVLPs containing flagellin or LTB are faster and stronger than the response to sRVLP.

To further understand how IgG subclasses are affected by the presence of membrane-anchored flagellin or LTB, the RABV-specific IgG, IgG1, IgG2a, IgG2b, IgG3, and IgM responses were determined by ELISA. The total IgG response (**Figure 2B**) induced by cRVLPs was notably higher than the response induced by EVLP. As shown in **Figures 2C,D** three VLPs promoted the oxproduction of IgG1 and IgG2a subtypes, but the IgG1 responses induced by EVLP-L were significantly stronger than those in response to EVLP or EVLP-F. Moreover, EVLP-F induced the highest IgG2a response among the three groups. As shown in **Figure 2H**, the ratios of IgG1 to IgG2a in the groups immunized with EVLP and EVLP-F were significantly lower than EVLP-L. Furthermore, cRVLPs induced higher levels of IgG2b (**Figure 2E**), IgG3 (**Figure 2F**), and IgM (**Figure 2G**) compared with EVLP. These results demonstrate that cRVLPs containing flagellin or LTB improved antibody production and produced different subclasses of antibodies. EVLP-F induced an IgG2a-dominant response, whereas EVLP-L induced an IgG1-dominant response, indicating different activation mechanisms in B cells.

### cRVLPs CONTAINING FLAGELLIN OR LTB ELICIT ENHANCED CELL-MEDIATED IMMUNE RESPONSES IN MICE

**FIGURE 3:**
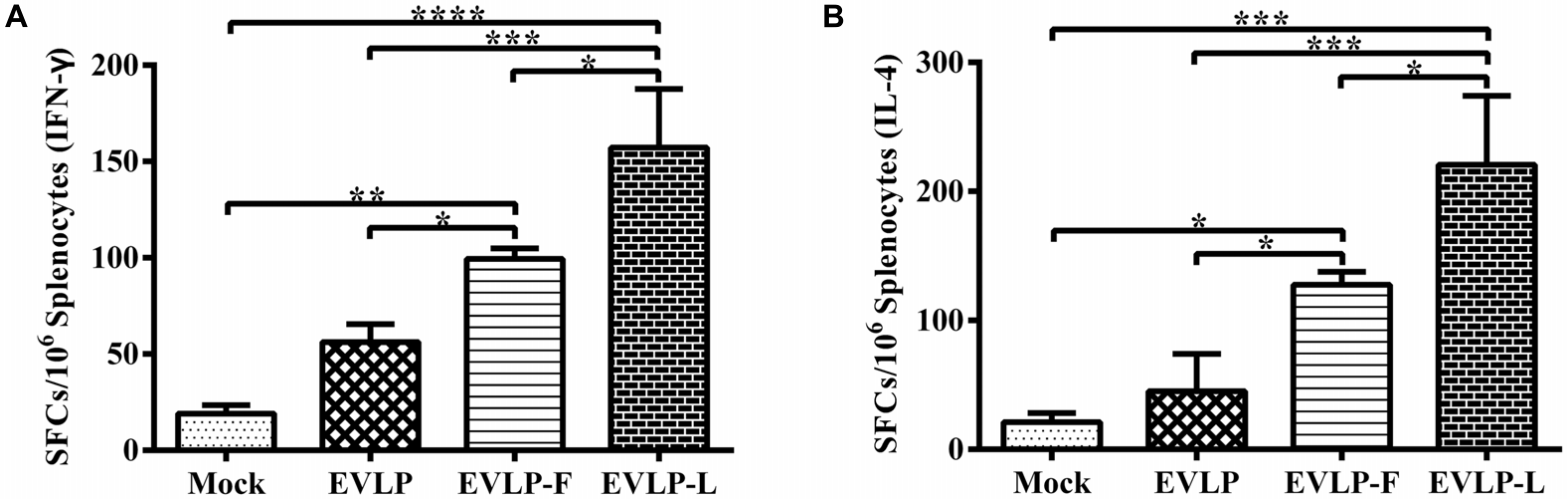
**Enzyme-linked immunospot (ELISpot) analyses of IFN-γ and IL-4 secretion in mice.** Splenocytes from mice from the four groups were stimulated with inactivated ERA 2 weeks after the second immunization. Splenocytes producing IFN-γ **(A)** or IL-4 **(B)** were identified using ELISpot kits. The data represent the mean numbers and SDs of SFCs per million splenocytes from three mice in each group and were analyzed by one-way ANOVA (*P < 0.05, **P < 0.01, ***P < 0.001, ****P < 0.0001).

We further evaluated the cell-mediated immune responses using IFN-γ and IL-4 ELISpot assays. The results from the IFN-γ ELISpot assay are shown in **Figure 3A**. The SFCs produced from the splenocytes of mice immunized with EVLP-F or EVLP-L were significantly higher compared with mice immunized with EVLP. As shown in **Figure 3B**, immunization with EVLP-F or EVLP-L also resulted in increased IL-4 responses, with SFCs production that were significantly higher than that following immunization with EVLP. These results demonstrate that membrane-anchored flagellin or LTB incorporated into sRVLP enhances IFN-γ and IL-4 responses in mice.

**FIGURE 4:**
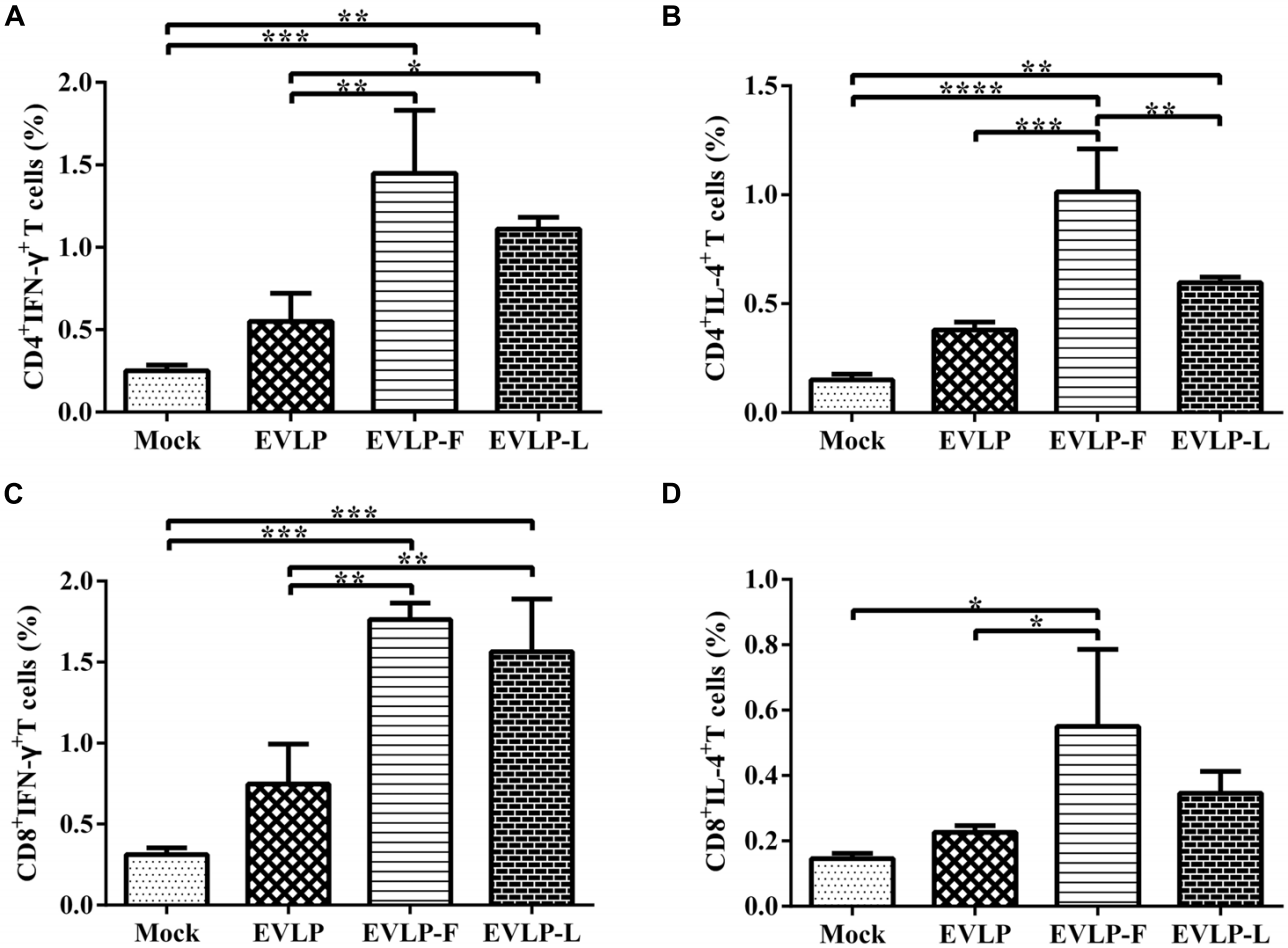
**Intracellular cytokine staining (ICS) analysis of CD4^**+**^ and CD8^**+**^ T cells from mice producing IFN-γ or IL-4.** Splenocytes from three mice in each group 2 weeks after the second immunization were cultured and stimulated with inactivated ERA. The splenocytes were then stained with mouse anti-CD4, anti-CD8, anti-IFN-γ, and anti-IL-4 monoclonal antibodies. CD4^+^ T cells producing IFN-γ **(A)** or IL-4 **(B)** and CD8^+^ T cells producing IFN-γ **(C)** or IL-4 **(D)** are shown. The data represent the means and SDs of double-positive cell percentages and were analyzed by one-way ANOVA (*P < 0.05, **P < 0.01, ***P < 0.001, ****P < 0.0001).

To characterize the T cell responses induced by cRVLPs, we further analyzed the ability of these cRVLPs to induce IFN-γ- or IL-4-secreting CD4^+^ and CD8^+^ T cells using intracellular cytokine staining (ICS). Compared with EVLP, cRVLPs induced significantly more IFN-γ-secreting CD4^+^ or CD8^+^ T cells, and there was no significant difference between mice immunized with EVLP-F and EVLP-L (**Figures 4A,C**). As shown in **Figures 4B,D**, the percentage of IL-4-secreting CD4^+^ or CD8^+^ T cells in mice vaccinated with EVLP-F or EVLP-L was higher than in the EVLP group, and the difference between EVLP-F and EVLP was significant. These results suggest that the use of membrane-anchored flagellin or LTB as adjuvant molecules to modify sRVLP markedly improves CD4^+^ and CD8^+^ T cell responses in mice.

### IMMUNIZATION WITH cRVLPs RECRUITS AND/OR ACTIVATES MORE B CELLS AND DCs

**FIGURE 5:**
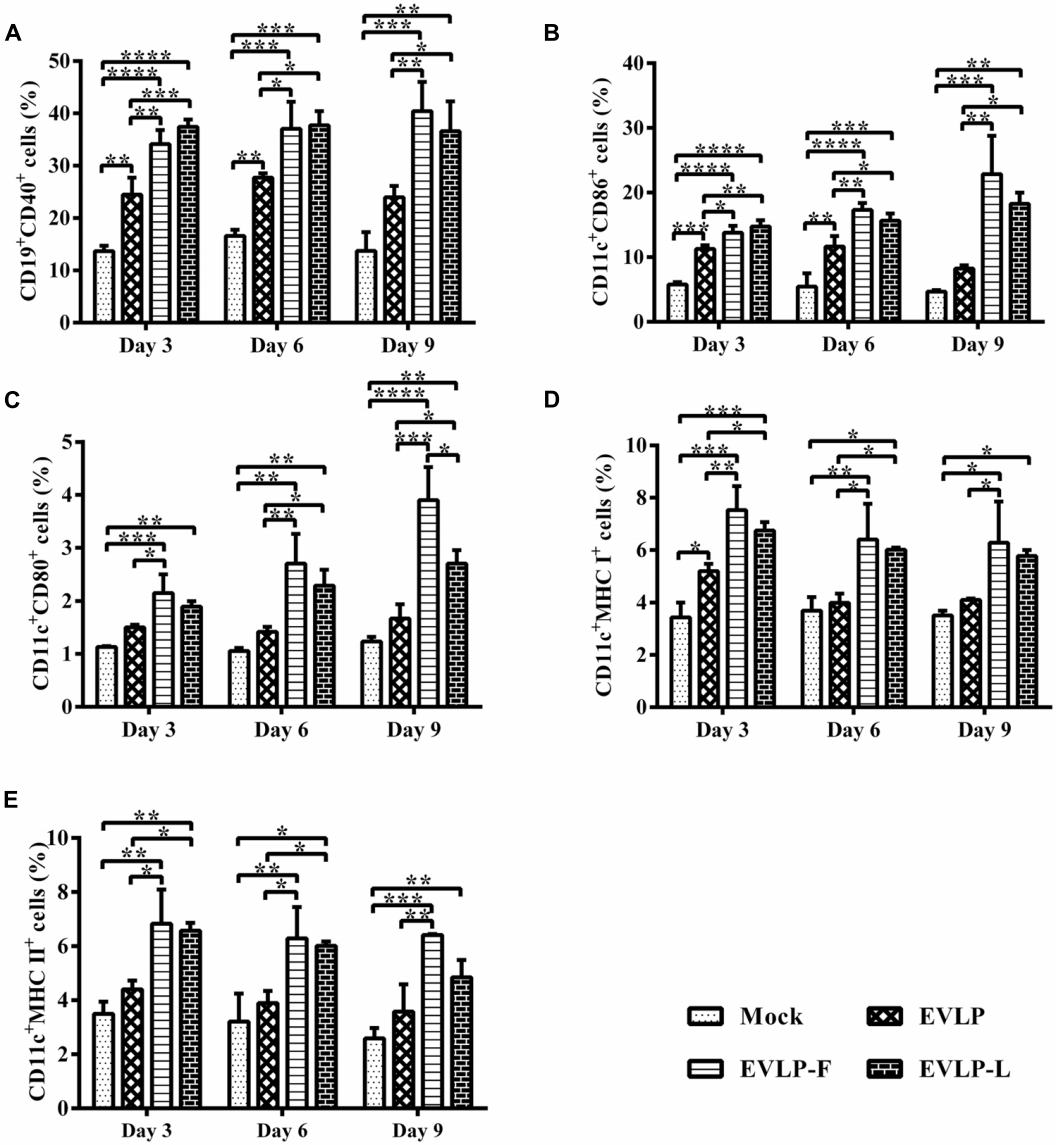
**Flow cytometry assay for recruitment and/or activation of (B) cells and DCs in lymph nodes from mice.** Inguinal lymph nodes cells were isolated from 3 mice from each group on days 3, 6, and 9 after the first immunization and were stained with mouse anti-CD19, anti-CD40, anti-CD11C, anti-CD80, anti-CD86, anti-MHC I, and anti-MHC II monoclonal antibodies. The double-positive CD19^+^CD40^+^ **(A)**, CD11c^+^CD86^+^ **(B)**, CD11c^+^CD80^+^ **(C)**, CD11c^+^MHC I^+^ **(D)**, and CD11c^+^MHC II^+^ **(E)** cells were plotted. The data represent the means and SDs of double-positive cell percentages and were analyzed by one-way ANOVA (*P < 0.05, **P < 0.01, ***P < 0.001, ****P < 0.0001).

To investigate whether cRVLPs recruit and/or activate a larger number of B cells and DCs than does EVLP, the percentages of B cells and DCs in lymph nodes were analyzed by flow cytometry (CD19 and CD40 for B cells; CD11c, CD86, CD80, MHC I, and MHC II for DCs). As shown in **Figure 5A**, after the first dose, more activated B cells were detected in the immunized groups than in the control group. Importantly, the percentages of CD19^+^CD40^+^ cells in lymph nodes from mice vaccinated with EVLP-F or EVLP-L were significantly higher at all time points after primary immunization than among mice vaccinated with EVLP. Moreover, no decreasing trend was observed in the expression levels of CD19 and CD40 in lymph node cells from the EVLP-F or EVLP-L group on day 9 compared with days 3 or 6.

**Figure 5B** shows that a greater number of DCs were detected in the inguinal lymph nodes of mice injected with the three rabies VLPs compared with mice treated with PBS. Moreover, the percentage of CD11c and CD86 double-positive cells in the EVLP-F or EVLP-L group was significantly higher than in the EVLP group at all time points. By day 9 after the first dose, the expression levels of CD11c and CD86 decreased in the EVLP group but remained elevated in the EVLP-F and EVLP-L groups. As shown in **Figure 5C**, there was an expansion of CD11c^+^ and CD80^+^ cells in lymph nodes from mice vaccinated with EVLP-F or EVLP-L on days 3, 6, and 9 after the primary immunization. No differences were observed between the EVLP and control groups; however, the expression of CD11c and CD80 in the cRVLPs groups was significantly higher on days 6 and 9 compared with mice immunized with PBS or EVLP. cRVLPs also induced stronger expression of MHC I and MHC II on DCs than did sRVLP. These differences reached statistical significance on days 3 and 6, and the difference between the EVLP-F and EVLP groups persisted at days 9 (**Figures 5D,E**). Collectively, our data suggest that cRVLPs containing membrane-anchored flagellin and LTB, respectively, recruit and/or activate more B cells and DCs in lymph nodes than do sRVLP while also inducing stronger expression of MHC I and MHC II on DCs.

### IMMUNIZATION WITH EVLP-F or EVLP-L PROTECTS MICE FROM LETHAL CHALLENGE WITH THE RABV STREET STRAIN

**FIGURE 6:**
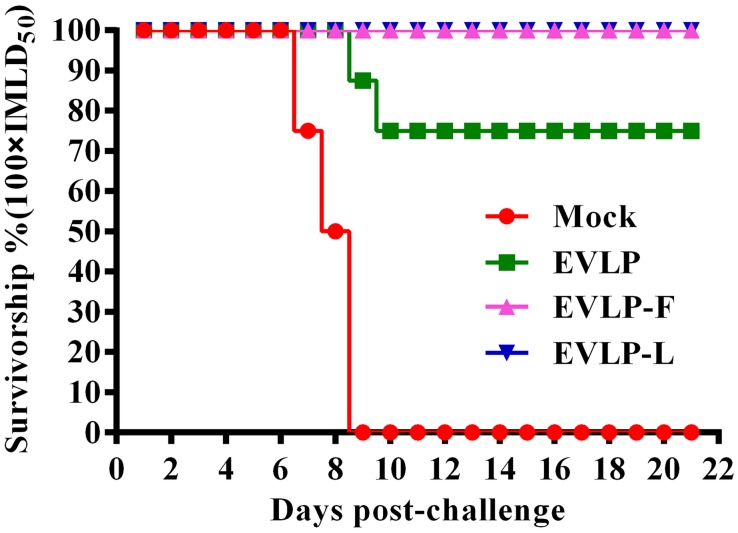
**Challenge test in mice.** Mice from the four groups were challenged i.m. 4 weeks after the second vaccination with 100 × IMLD_50_ (*n* = 8) of the RABV street strain HNPB3 and observed for 21 days. The percentages of surviving mice in the different groups at different time points after challenge were recorded.

To investigate whether the immune responses induced by cRVLPs could protect mice against challenge with RABV, all mice in the four groups were inoculated via the i.m. route with 100× IMLD_50_ of HuNPB_3_ and were monitored daily for 21 days. As shown in **Figure 6**, all mice vaccinated with EVLP-F or EVLP-L survived a lethal challenge with RABV and showed no symptoms of rabies during the 21-day observation period. However, 2 of the mice immunized with EVLP showed clinical signs of rabies and died within 10 days; therefore, the percent survival was only 75%. All mice in the PBS-treated group died of exposure to RABV. RABV antigens were detected in the brain tissues of all mice euthanized following challenge. These results demonstrate that cRVLPs containing membrane-anchored flagellin or LTB are highly immunogenic and are efficacious in mice against lethal challenge with a high dose of the RABV street strain.

### ENHANCED ANTIBODY RESPONSES INDUCED BY EVLP-F OR EVLP-L IN DOGS

**FIGURE 7:**
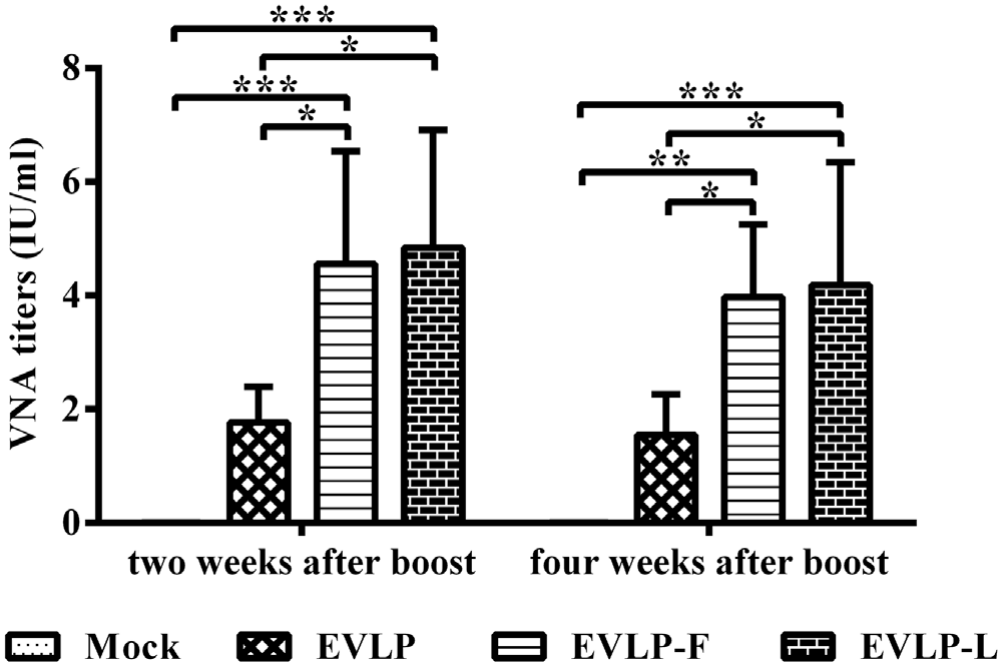
**Specific anti-RABV VNA responses in dogs.** Dogs were vaccinated i.m. twice with EVLP, EVLP-F, EVLP-L, or PBS at 2-week intervals. Blood was collected 2 and 4 weeks after the second dose. VNA titers were measured by FAVN. The data represent the means ± SD for five dogs from each group and were analyzed by one-way ANOVA (*P < 0.05, **P < 0.01, ***P < 0.001).

To evaluate the immunogenicity of cRVLPs in dogs, which were immunized with EVLP, EVLP-F, or EVLP-L, and VNA titers were measured. As shown in **Figure 7**, VNA titers in dogs immunized with EVLP-F or EVLP-L were significantly higher than in dogs treated with EVLP. Two weeks after the second vaccination, the mean VNA titers in dogs immunized with EVLP, EVLP-F, and EVLP-L were 1.76, 4.56, and 4.85 IU/ml, respectively. Four weeks after the boost immunization, the mean VNA titers in the three experimental groups were 1.54, 3.97, and 4.18 IU/ml, respectively. No significant difference was observed between VNA titers from the EVLP-F and EVLP-L groups. These data suggest that EVLP-F and EVLP-L are immunogenic in dogs and can induce higher specific anti-RABV VNA responses than can sRVLP.

### ENHANCED CELLULAR IMMUNE RESPONSES INDUCED BY EVLP-F OR EVLP-L IN DOGS

**FIGURE 8:**
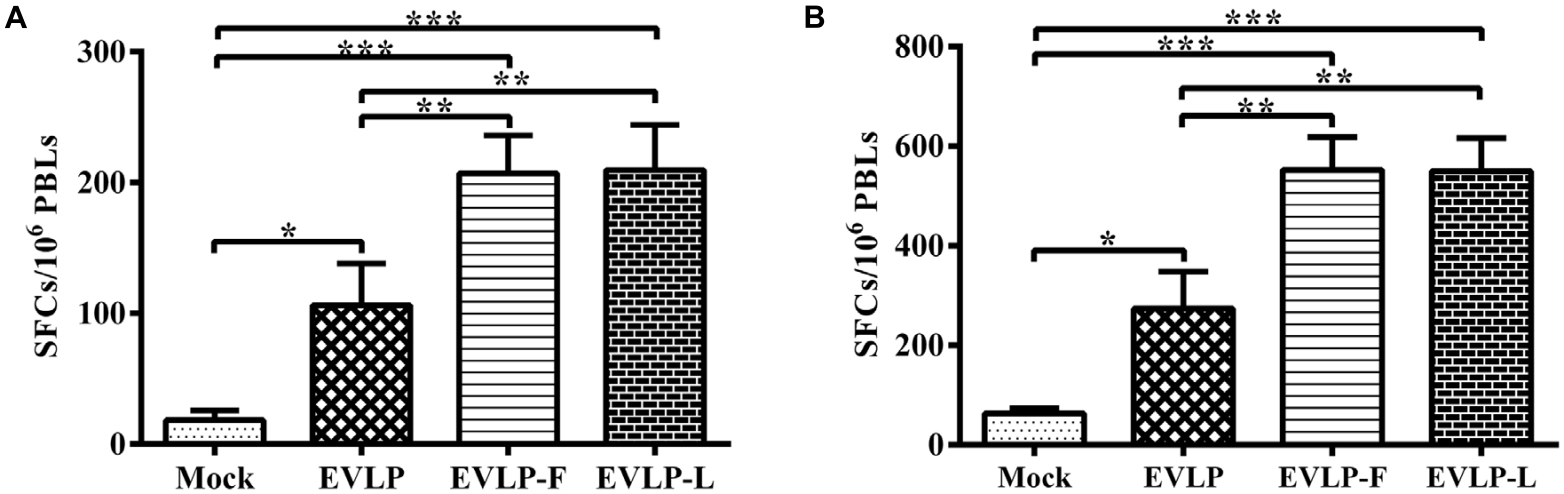
**Enzyme-linked immunospot assays of IFN-γ and IL-4 secretion in dogs.** PBMCs were isolated from dogs in each group and were stimulated with inactivated ERA. The PMBCs producing IFN-γ **(A)** or IL-4 **(B)** were identified using ELISpot kits. The data represent the mean numbers and SDs of SFCs per million PMBCs from 3 dogs in each group and were analyzed by one-way ANOVA (*P < 0.05, **P < 0.01, ***P < 0.001).

To further assess cellular immune responses in dogs following vaccination with cRVLPs, we determined IFN-γ and IL-4 secretion using ELISpot assays. **Figure 8A** shows that the SFCs representing IFN-γ secretion by PBMCs in dogs vaccinated with EVLP-F or EVLP-L were significantly higher than in dogs immunized with EVLP at the same dosage. As shown in **Figure 8B**, a significantly higher number of SFCs representing IL-4 secretion were detected in PBMCs from dogs immunized with cRVLPs compared with dogs injected with EVLP. No significant difference in the ELISpot assays was observed between the EVLP-F and EVLP-L. Overall, these results suggest that EVLP-F and EVLP-L can induce remarkably increased specific cellular immune responses in dogs compared with sRVLP.

## DISCUSSION

A wide variety of VLP-based vaccine candidates have been generated in different expression systems. Some of them have entered clinical development, and a few have been licensed and commercialized. Molecular adjuvants have been used in the VLPs’ research. The adding of immunostimulatory signals to VLP would enhance their efficacy. CD40L-containing HIV VLP showed great capacity to activate DCs *in vitro*, and also proved to be immunogenic in mice in raising both humoral and cellular responses to HIV-1 Gag (Franco et al., 2011). Immunized with chimeric SIV VLP containing glycosylphosphatidylinositol (GPI)-anchored GM-CSF, which induced significantly higher levels of Env-specific antibodies and neutralizing activity than those induced by standard SIV VLP or standard SIV VLP mixed with soluble GM-CSF (Skountzou et al., 2007). Therefore, to further improve the immunogenicity of sRVLP and to develop a more effective rabies vaccine candidate, we modified the sRVLP with immunostimulatory molecule to enhance the immune responses against rabies. In the present study, we constructed and generated two cRVLPs that contain G and M of RABV ERA, and membrane-anchored flagellin or LTB to act as a molecular adjuvant. These cRVLPs induced enhanced humoral and cellular immune responses to RABV by i.m. administration in mice and dogs, and provided complete protection against lethal challenge with the RABV street strain in a mouse model.

Flagellin is a TLR5 agonist that activates the innate immune response, which is considered important for priming and regulating the adaptive immune response, subsequently resulting in detectable serum antibody responses. In this study, EVLP-F containing membrane-anchored flagellin induced faster and stronger RABV-specific antibody responses in mice and dogs than did EVLP. Membrane-anchored flagellin retains its adjuvant activity, with the fusion form producing a strong immunoenhancement effect that resulted in higher VNA titers. The Ig isotype profile reflected a different mechanism of antigen presentation and cognate interaction with helper T cells (Wang et al., 2010). EVLP-F induced significantly enhanced IgG2a responses, which are responsible for protection against encapsulated pathogens (Visciano et al., 2012). The relatively low IgG1/IgG2a ratio demonstrated that EVLP-F elicited a Th1-preferred Th1/Th2 response profile that was preferentially involved in cell-mediated immunity. Splenocytes from mice immunized with EVLP-F produced stronger IFN-γ and IL-4 responses, representing secretion of both Th1- and Th2-type cytokines. However, serological tests did not reveal significant IgG1 responses in mice vaccinated with EVLP-F. These results suggest that IgG2a dominates the response through a cytokine-independent mechanism. IgG1 and IgG3 mediate important protective biological functions, such as complement fixation, opsonization, and induction of antibody-dependent cell cytotoxicity (Visciano et al., 2012). Determining whether the high levels of IgG1 and IgG3 induced by EVLP-F perform any of these actions requires further investigation. During the immune responses induced by infection or vaccination, the first antibody produced is IgM, which possesses a high valency due to its pentameric structure (Klimovich, 2011). In this case, the notably enhanced IgM response induced by EVLP-F may help to prevent the spread of RABV from invasion sites to the CNS while higher-affinity IgG antibodies are being formed in germinal centers (Dorfmeier et al., 2013).

Previous studies did not observe the expression of TLR5 on the surface of murine B cells or the activation of IL-1R-associated kinase 1 by flagellin (Mizel and Bates, 2010). However, in this study, the EVLP-F containing flagellin recruited and/or activated a significantly higher number of B cells in draining lymph nodes following immunization. In light of this result, the use of flagellin as an adjuvant for immunization produces a marked effect on B cell activation, although additional research into the mechanism is necessary for confirmation. It is accepted that most adjuvants (especially TLR agonists) induce DCs maturation, activation, and migration to secondary lymphoid sites (Mizel and Bates, 2010). EVLP-F promoted a notable increase in the recruitment and/or activation of DCs to draining lymph nodes and also stimulated higher expression levels of MHC I and MHC II on DCs compared with EVLP. These results demonstrated that EVLP-F could be more efficiently taken up by DCs, which is consistent with antigen processing and presentation due to the high levels of MHC II molecules. This increased expression, accompanied by the production of co-stimulatory molecules and secretion of cytokines, results in enhanced immune responses against RABV. Membrane-anchored flagellin induced higher levels of MHC I on DCs; thus, EVLPs-F may be more efficiently processed in the cytosol of DCs and presented by MHC I molecules. Finally, cross-presentation to cytotoxic CD8^+^ Th cells could induce potent cytotoxic immunity (Win et al., 2011).

The ability of flagellin to promote robust CD4^+^ T cell-dependent responses has been reported. However, less is known about whether flagellin affects the CD8^+^ T cell responses. Some researchers (Cuadros et al., 2004; Huleatt et al., 2007; Braga et al., 2010) have reported that flagellin has a significant effect on CD8^+^ T cell activation, whereas other studies (Schwarz et al., 2003; Didierlaurent et al., 2004) did not observe a significant effect. In our study, EVLP-F induced significantly stronger responses in antigen-specific IFN-γ- or IL-4-secreting CD4^+^ T and CD8^+^ T cells compared with immunization with sRVLP. These results demonstrated that the membrane-anchored flagellin incorporated into cRVLPs significantly induced and promoted activation of both CD4^+^ T and CD8^+^ T cells; this activation resulted in the induction of enhanced CD4^+^ T cell-dependent humoral responses and CD8^+^ T cell-mediated immune responses, both of which play important roles in viral clearance during viral infection.

Heat-labile enterotoxin B subunit has been extensively exploited as a mucosal adjuvant in animal models. However, the use of LTB as a parenteral adjuvant is rare. In this study, we evaluated the immune responses induced by i.m. immunization of mice with EVLP-L containing membrane-anchored LTB. As observed with EVLP-F, significantly higher VNA titers and stronger IgG, IgG2b, IgG3, and IgM responses were measured in mice vaccinated with EVLP-L compared with EVLP. In contrast with EVLP-F, EVLP-L induced a predominant IgG1 response, resulting in a relatively high IgG1/IgG2a ratio. LTB induced a mixed Th1–Th2 immune response when administered via the oral route (Nakagawa et al., 1996; Takahashi et al., 1996) and a Th1-biased immune response when immunized via the intranasal route (Conceicao et al., 2006). Moreover, membrane-anchored LTB incorporated into sRVLP and administered i.m. induced enhanced humoral immune responses with a Th2-preferred Th1/Th2 response profile, which is more effective for the regulation and support of B-cell responses. Thus, the route of immunization is an important factor in the type of immune response induced by immunization using LTB as an adjuvant.

The interaction between LTB and the GM1 receptor activates DCs and other APCs and significantly strengthens the presentation of LTB-conjugated antigens. Furthermore, GM1-mediated antigen presentation enhances the proliferation of and cytokine expression by antigen-specific CD4^+^ T cells, providing helper signals for the induction of antigen-specific B cells. All of the above responses contribute to the activation of B cells and CD4^+^ T cells (Nashar et al., 2001; Ertl, 2009; Wang et al., 2012). LTB was incorporated into sRVLP as a membrane-anchored subunit and retained its strong adjuvant activity, which stimulated the maturation, activation, and/or recruitment of more B cells and DCs in draining lymph nodes. The secretion of antigen-specific IFN-γ and IL-4 cytokines by splenocytes was remarkably increased in mice immunized with EVLP-L. Moreover, EVLP-L induced significantly enhanced antigen-specific IFN-γ- or IL-4-secreting CD4^+^ T and CD8^+^ T cells responses.

Overall, the cRVLPs containing membrane-anchored flagellin or LTB induced significantly enhanced humoral and cellular immune responses compared to sRVLPs in mice immunized via the i.m. route. And the enhanced immune responses provided complete protection against a lethal challenge with a high dose of RABV in mice. Dogs represent a sizeable animal population that requires vaccination for rabies control (Ge et al., 2011), especially in developing countries where dogs serve as vector for rabies transmission. Immunization of dogs with EVLP-F and EVLP-L elicited high specific anti-RABV VNA titers and enhanced antigen-specific IFN-γ and IL-4 cytokine secretion by PBMCs.

Together, these results demonstrate that membrane-anchored flagellin and LTB possess strong adjuvant activity when incorporated into sRVLP. EVLP-F and EVLP-L can trigger an innate immune response and then rapidly elicit RABV-specific humoral and cellular immune responses. Therefore, they can be developed as a safe and more efficacious animal rabies vaccine candidate. Moreover, flagellin and LTB as powerful mucosal adjuvants have been widely used in mucosal immunity. So further investigation is required to examine whether cRVLPs containing membrane-anchored flagellin or LTB administered via the oral route enhance mucosal and systemic immune responses against RABV. Such cRVLPs may perhaps be developed as an effective oral vaccine for the prevention and control of rabies in animals.

## Conflict of Interest Statement

The authors declare that the research was conducted in the absence of any commercial or financial relationships that could be construed as a potential conflict of interest.
